# Enhanced recovery after surgery for microsurgical oblique lateral interbody fusion: A retrospective before-and-after cohort study

**DOI:** 10.1097/MD.0000000000051016

**Published:** 2026-10-09

**Authors:** Jie Chen, Huaxi Li, Lingyun Qiu, Xinghui Xie, Xia Qin

**Affiliations:** a Department of Orthopedics, Xinqiao Hospital, Army Medical University, Chongqing, People’s Republic of China.

**Keywords:** enhanced recovery after surgery, length of stay, lumbar degenerative disease, oblique lateral interbody fusion, perioperative care, retrospective cohort

## Abstract

This study evaluates the real-world effectiveness and safety of a standardized enhanced recovery after surgery (ERAS) pathway in patients undergoing single-level microsurgical oblique lateral interbody fusion (MIS-OLIF) for lumbar degenerative disease. This retrospective before-and-after cohort study included 89 patients who underwent single-level MIS-OLIF at L3-4 or L4-5 between January 2021 and December 2023. Patients were categorized according to the institutional perioperative pathway in effect at the time of surgery: conventional care (n = 44) or ERAS (n = 45). The primary outcome was postoperative length of stay. Secondary outcomes included back-pain and leg-pain visual analog scale (VAS) scores, Oswestry Disability Index (ODI), ERAS-component adherence, discharge readiness, complications, readmission, and patient satisfaction. Selection bias was addressed using propensity-score overlap weighting, and repeated VAS and ODI measurements were analyzed using linear mixed-effects models. The ERAS group had a shorter unadjusted hospital stay than the conventional-care group (6.2 ± 0.8 vs 7.5 ± 1.2 days). After overlap-weighting adjustment, ERAS was associated with an adjusted reduction in postoperative length of stay of −1.1 days (95% confidence interval [CI]: −1.4 to −0.8; *P* < .001). Early postoperative back and leg pain were significantly lower in the ERAS group (group-by-time interaction *P* = .02 and *P* = .04, respectively). Overall ERAS adherence was 94.7% (median). Complication rates were 11.1% vs 18.2% (adjusted odds ratio 0.52, 95% CI: 0.14–1.94, *P* = .33), and satisfaction rates (satisfied/very satisfied) were 97.8% vs 93.2% (*P* = .36). In this single-center real-world cohort, implementation of a standardized ERAS pathway was associated with earlier discharge after single-level MIS-OLIF. The effects on pain, function, satisfaction, and safety should be interpreted according to the adjusted analyses and remain subject to residual confounding inherent in the retrospective before-and-after design.

## 1. Introduction

Enhanced recovery after surgery (ERAS) is an evidence-based, multidisciplinary approach designed to reduce perioperative physiological and psychological stress, standardize care, and accelerate recovery. Rather than representing a single intervention, ERAS combines patient education, optimized fasting and nutrition, multimodal analgesia, rational fluid and catheter management, early oral intake, and early mobilization across the preoperative, intraoperative, and postoperative phases.^[1,2]^ In spine surgery, ERAS pathways have been associated with shorter hospitalization and improved perioperative efficiency, although the effects vary according to surgical procedure, patient population, and protocol adherence.^[3-14]^

Oblique lateral interbody fusion (OLIF) uses an anterior-to-psoas retroperitoneal corridor and preserves the posterior paraspinal musculature and ligamentous structures. For appropriately selected patients with degenerative lumbar disease, OLIF may reduce tissue disruption and blood loss while achieving indirect neural decompression and interbody reconstruction.^[15-20]^ Despite the minimally invasive nature of the approach, postoperative pain, delayed mobilization, gastrointestinal recovery, catheter management, and discharge coordination remain clinically relevant.

Previous studies have reported ERAS pathways for lumbar fusion and other spinal procedures.^[6-9]^ Therefore, merely demonstrating that ERAS is “effective” provides limited novelty. The clinically relevant unanswered questions are whether a multidisciplinary pathway can be implemented reliably in routine microsurgical oblique lateral interbody fusion (MIS-OLIF) practice, whether the groups are comparable after accounting for temporal and baseline differences, which pathway elements are actually completed, and whether earlier discharge is achieved without compromising safety.

Accordingly, this study evaluated the real-world implementation of a standardized ERAS pathway in patients undergoing single-level MIS-OLIF. The primary objective was to compare postoperative length of stay (LOS) between ERAS and conventional care after adjustment for measured confounding. Secondary objectives were to compare early pain and functional recovery, quantify adherence to individual ERAS components, and evaluate patient satisfaction and perioperative safety.

## 2. Materials and methods

### 2.1. Study design, setting, and ethics

This was a single-center retrospective before-and-after cohort study of patients undergoing MIS-OLIF between January 2021 and December 2023 at Xinqiao Hospital, Army Medical University. The study was approved by the Medical Ethics Committee of the Second Affiliated Hospital of Army Medical University (approval date: August 5, 2024; approval number: 2024-R.No.235-01) and was conducted in accordance with the Declaration of Helsinki.

The manuscript was prepared in accordance with the principles of transparent reporting for observational cohort studies.

### 2.2. Participants

The hospital records of patients treated with single-level MIS-OLIF during the study period were reviewed. Eligible diagnoses included degenerative spondylolisthesis, lumbar spinal stenosis with instability, and symptomatic lumbar segmental instability at L3-4 or L4-5. Inclusion criteria were: age ≥ 18 years; a single symptomatic level at L3-4 or L4-5; failure of appropriate nonoperative treatment for at least 3 months; and completion of MIS-OLIF by the study surgical team.

Exclusion criteria were multilevel lumbar degenerative disease, an operative level at L5-S1, severe cardiovascular or cerebrovascular disease precluding the standardized pathway, psychiatric or cognitive impairment preventing reliable communication or outcome assessment, revision surgery, osteoporosis with T-score ≤ −2.5, and active infection. A total of 112 patients were screened; 23 were excluded (12 for L5-S1 level, 6 for multilevel disease, 3 for severe comorbidity, 2 for cognitive impairment). The remaining 89 patients were consecutively enrolled.

Patients undergoing L5-S1 surgery were excluded to reduce technical and anatomical heterogeneity. Although an oblique/anterior approach can be used at L5-S1, access at this level requires a distinct lumbosacral vascular corridor and differs from conventional prepsoas OLIF at L3-4 and L4-5. The present findings should therefore not be generalized to L5-S1 OLIF.^[21]^

### 2.3. Group definition and control of protocol assignment

Patients were categorized according to the institutional perioperative pathway in effect at the time of surgery. The conventional-care group comprised 44 patients treated before formal ERAS implementation (between January 2021 and June 2022), whereas the ERAS group comprised 45 patients treated after implementation of the standardized pathway (between August 2022 and December 2023). A transition period (July 2022) was used for staff training and protocol refinement; patients treated during this period were excluded from analysis. Protocol assignment was therefore determined by calendar period rather than by individual patient or clinician preference.

Both groups underwent MIS-OLIF performed by the same senior spine surgeon. Surgical indications and the core operative technique remained unchanged across the study period. Implant type (polyetheretherketone cages with autograft), fixation strategy (bilateral pedicle screw-rod), anesthesia practice (total intravenous anesthesia with propofol and remifentanil), and postoperative ward resources did not materially change over time.

### 2.4. Multidisciplinary ERAS development and implementation

The ERAS pathway was developed by a multidisciplinary team including spine surgeons, anesthesiologists, ward nurses, nutrition personnel, and rehabilitation staff. Before implementation (June to July 2022), the team standardized patient-education materials, perioperative medication orders, nutritional instructions, catheter and drain management, mobilization milestones, documentation forms, and discharge planning. Relevant staff received pathway training during the transition month.

To assess implementation fidelity and possible cross-contamination, completion of each pathway component was determined from medication administration records, nursing documentation, diet orders, catheter and drainage records, and rehabilitation records. Each component was coded as completed, not completed, contraindicated, or undocumented. Overall adherence was calculated as the number of completed eligible components divided by the number of eligible components for each patient. Component-level data were retrospectively extractable for all planned elements.

### 2.5. Perioperative pathways

The intervention was evaluated as a bundled multidisciplinary pathway. The study was not designed to establish the independent causal effect of any individual component. Table 1 lists the operational definition of each component and the actual patient-level adherence data.

**Table 1:** Perioperative pathway components and actual protocol adherence.

Component	ERAS pathway	Conventional care	Actual completion, n/N (%)
Preoperative education	Structured ERAS booklet and face-to-face education covering pain control, nutrition, mobilization, rehabilitation, and discharge planning	Routine perioperative education	ERAS: 43/45 (95.6) Control: 20/44 (45.5)
Fasting and nutrition	Solid food/nutritional supplement completed 6 h before surgery; clear nutritional liquid completed 2 h before anesthesia, when not contraindicated	Traditional fasting plan: states 10 h fasting	ERAS: 41/45 (91.1) Control exposure: 0/44
Preemptive analgesia	Celecoxib 200 mg and pregabalin 150 mg orally 2 h before anesthesia induction	No standardized preemptive regimen	ERAS: 40/45 (88.9) Control exposure: 0/44
Antibiotic prophylaxis	Cefuroxime 1.5 g intravenously within 30 min before incision	Cefuroxime 1.5 g intravenously within 30 min before incision	ERAS: 45/45 (100) Control: 44/44 (100)
Intraoperative management	Forced-air warming (maintain core temp > 36°C), goal-directed fluid therapy (stroke volume variation < 12%), dexamethasone 10 mg IV for antiemesis	Conventional anesthetic and fluid management	ERAS: 44/45 (97.8) Control: N/A
Postoperative multimodal analgesia	Piroxicam 40 mg intravenously every 12 h for 3 d; celecoxib 200 mg orally every 12 h from postoperative day 4; pregabalin 75 mg every 12 h; tramadol 0.1 g intramuscularly as rescue analgesia	Analgesia based on the conventional physician-directed regimen	ERAS: 42/45 (93.3) Control: N/A
Early oral intake	Water after recovery from anesthesia and semi-liquid diet within 2 h, when clinically appropriate	Traditional postoperative dietary advancement	ERAS: 43/45 (95.6) Control: N/A
Urinary catheter	Removal after recovery from anesthesia (within 6–8 h post-surgery)	Removal according to conventional clinical judgment	ERAS: 42/45 (93.3) Control: N/A
Drain removal	Removal when wound drainage was <50 mL per 24 h	Removal according to conventional clinical judgment	ERAS: 44/45 (97.8) Control: N/A
Early mobilization	Ankle pumps and lower-limb isometric exercises after recovery from anesthesia; assisted sitting, standing, and/or walking on postoperative day 1	Bed rest for 1–3 d followed by conventional mobilization	ERAS: 45/45 (100) Control: N/A
Postdischarge rehabilitation	Structured lumbar and lower-limb exercise instructions and follow-up plan	Traditional rehabilitation instructions	ERAS: 43/45 (95.6) Control: 41/44 (93.2)

ERAS = enhanced recovery after surgery, IM = intramuscular, IV = intravenous, N/A = not applicable because not part of the conventional protocol.

### 2.6. Surgical procedure

All procedures were performed under general anesthesia by the same senior spine surgeon using a microsurgical oblique lateral retroperitoneal approach. The patient was placed in the right lateral decubitus position with the left side up. After fluoroscopic level confirmation, a 4-cm oblique skin incision was made, and the retroperitoneal space was entered through the anterior-to-psoas corridor. The target disc was identified, discectomy and endplate preparation were performed under microscopic visualization, and a polyetheretherketone cage filled with local autograft was placed. Supplemental posterior fixation with bilateral pedicle screws and rods was performed in the same prone position through a separate midline incision. No direct decompression was performed; indirect decompression was achieved by disc height restoration and ligamentotaxis. A closed suction drain was placed in the retroperitoneal space in all cases. The operative technique remained unchanged throughout the study period.

### 2.7. Outcomes and definitions

The primary outcome was postoperative LOS, defined as the number of calendar days from the date of surgery to hospital discharge. Total hospital LOS from admission to discharge was analyzed separately.

Secondary outcomes included back-pain visual analog scale (VAS), leg-pain VAS, Oswestry Disability Index (ODI), time to first oral intake, time to urinary-catheter removal, time to drain removal, time to first ambulation, rescue opioid use (tramadol doses), postoperative complications (graded by Clavien-Dindo), 30-day readmission, reoperation, and patient satisfaction.

### 2.8. Pain and functional assessment

Back pain and leg pain were assessed separately using an 11-point VAS ranging from 0 (no pain) to 10 (worst imaginable pain). Assessments were scheduled preoperatively and at 6, 12, 24, 48, and 72 hours and 7 days after surgery. The ODI (version 2.0) was recorded preoperatively and at 12 hours, 1 day, 3 days, and 7 days postoperatively.

In-hospital assessments were performed by trained research nurses using a standardized form. The 7-day assessment was completed during the outpatient follow-up visit. Assessors were not blinded to group assignment; this limitation is acknowledged in Section 4.

### 2.9. Patient satisfaction

Patient satisfaction was measured at the 7-day follow-up using an institutional single-item questionnaire with 5 response categories: very satisfied, satisfied, neutral, dissatisfied, and very dissatisfied. The questionnaire was administered by a research nurse during the outpatient visit. The prespecified satisfaction outcome was the proportion of patients reporting “satisfied” or “very satisfied.” The complete response distribution was retained for analysis.

### 2.10. Standardized discharge-readiness criteria

To ensure that length-of-stay comparisons reflected the time required to achieve comparable clinical readiness rather than different discharge thresholds, patients in both groups were considered eligible for discharge when all of the following criteria were met:

1. Stable vital signs and no active medical or surgical complication requiring inpatient treatment;
2. Pain adequately controlled with oral medication;
3. Ability to tolerate oral food and fluids without clinically significant nausea or vomiting;
4. Spontaneous urination after urinary catheter removal;
5. Safe independent ambulation or ambulation with the patient’s prescribed assistive device;
6. Ability to perform basic transfers and activities required for home living;
7. No progressive neurological deficit;
8. Surgical wound condition satisfactory, with drainage tube removed or a clearly documented outpatient drainage plan;
9. Completion of discharge education, medication reconciliation, rehabilitation instructions, and home-care planning.

### 2.11. Statistical analysis

Continuous variables were summarized as mean ± standard deviation or median (interquartile range), according to their distribution. Categorical variables were summarized as counts and percentages. Baseline balance was evaluated using standardized mean differences (SMDs), with an absolute SMD < 0.10 indicating acceptable balance; baseline comparability was not judged solely by null-hypothesis P values.

Because treatment assignment was nonrandomized and determined by implementation period, a propensity score was estimated using age, sex, body mass index (BMI), smoking status, American Society of Anesthesiologists (ASA) class, hypertension, diabetes, cardiovascular disease, previous lumbar surgery, primary diagnosis, operative level, baseline back-pain VAS, baseline leg-pain VAS, and baseline ODI. The primary analysis used overlap weighting to improve covariate balance while retaining the cohort. Positivity, extreme weights, and post-weighting SMDs were assessed. After overlap weighting, all SMDs were < 0.10.

Postoperative LOS was compared using weighted linear regression. Repeated back-pain VAS, leg-pain VAS, and ODI scores were analyzed with linear mixed-effects models including fixed effects for group, time, and the group-by-time interaction; baseline score and prespecified confounders were included as covariates, and patient was modeled as a random intercept. Model-based adjusted mean differences with 95% confidence intervals were reported. Multiplicity across post hoc time-point contrasts was controlled using the Holm method.

Binary safety outcomes were compared using Fisher exact test and, when event counts permitted, adjusted logistic regression. Satisfaction was analyzed using Fisher exact test for the dichotomized outcome (satisfied/very satisfied vs others). Missing data were minimal (<2% for any variable) and handled using complete-case analysis. All tests were 2-sided, and *P* < .05 was considered statistically significant. Analyses were performed using R version 4.3.1 (R Foundation for Statistical Computing, Vienna, Austria).

## 3. Results

### 3.1. Participant flow and group allocation

A total of 112 patients were screened; 23 were excluded (12 for L5-S1 level, 6 for multilevel disease, 3 for severe comorbidity, 2 for cognitive impairment). The remaining 89 patients were consecutively enrolled: 44 in the conventional-care group (treated January 2021 to June 2022) and 45 in the ERAS group (treated August 2022 to December 2023). No patients were lost to follow-up for in-hospital outcomes; all 89 completed the 7-day assessment.

### 3.2. Baseline characteristics and covariate balance

Baseline demographic and clinical characteristics are presented in Table 2. Before weighting, the groups were well balanced on most characteristics, with all unweighted SMDs < 0.17. After overlap weighting, all SMDs were < 0.10, indicating excellent covariate balance.

**Table 2:** Baseline demographic and clinical characteristics.

Characteristic	ERAS (n = 45)	Conventional care (n = 44)	Unweighted SMD	Adjusted SMD
Age, yr	53.1 ± 3.2	52.6 ± 4.1	0.14	0.05
Female sex, n (%)	25 (55.6)	25 (56.8)	0.025	0.02
BMI, kg/m^2^	26.2 ± 3.5	25.8 ± 3.9	0.10	0.04
Current smoker, n (%)	9 (20.0)	12 (27.3)	0.17	0.06
ASA class III, n (%)	11 (24.4)	13 (29.5)	0.12	0.04
Hypertension, n (%)	15 (33.3)	14 (31.8)	0.03	0.01
Diabetes mellitus, n (%)	8 (17.8)	9 (20.5)	0.07	0.03
Cardiovascular disease, n (%)	6 (13.3)	7 (15.9)	0.08	0.02
Previous lumbar surgery, n (%)	4 (8.9)	5 (11.4)	0.08	0.03
Primary diagnosis: degenerative spondylolisthesis, n (%)	22 (48.9)	20 (45.5)	0.07	0.02
Primary diagnosis: stenosis with instability, n (%)	15 (33.3)	16 (36.4)	0.06	0.02
Primary diagnosis: segmental instability/DDD, n (%)	8 (17.8)	8 (18.2)	0.01	0.01
Operative level L3-4/L4-5, n	10/35	8/36	0.105	0.04
Baseline back-pain VAS	6.5 ± 1.8	6.7 ± 2.0	0.10	0.04
Baseline leg-pain VAS	5.8 ± 2.1	6.0 ± 2.2	0.09	0.03
Baseline ODI	48.2 ± 11.5	49.5 ± 12.0	0.11	0.05

Data are mean ± SD or n (%).

ASA = American Society of Anesthesiologists, BMI = body mass index, DDD = degenerative disc disease, ERAS = enhanced recovery after surgery, ODI = Oswestry Disability Index, SD = standard deviation, SMD = standardized mean difference, VAS = visual analog scale.

### 3.3. ERAS implementation and protocol adherence

Completion rates for the individual ERAS elements and exposure to similar elements in the conventional-care group are summarized in Table 1. Overall adherence in the ERAS group was 94.7% (median; IQR 88.9–100%), and 41 patients (91.1%) completed at least 80% of eligible components. The most consistently completed elements were antibiotic prophylaxis (100%), early mobilization (100%), and drain removal (97.8%), whereas the lowest adherence was observed for preemptive analgesia (88.9%) and early oral intake (95.6%). In the conventional-care group, no patient received the complete ERAS bundle, but cross-exposure to individual ERAS-like elements (mainly early mobilization and nonopioid analgesia) occurred in 7 patients (15.9%).

### 3.4. Length of stay and discharge readiness

The unadjusted mean postoperative hospital stay was 6.2 ± 0.8 days in the ERAS group and 7.5 ± 1.2 days in the conventional-care group (unadjusted difference −1.3 days, 95% confidence interval [CI]: −1.6 to −1.0; *P* < .001). After overlap weighting adjustment, ERAS was associated with an adjusted reduction of −1.1 days (95% CI: −1.4 to −0.8; *P* < .001). Total hospital LOS (from admission to discharge) was also significantly shorter in the ERAS group (8.5 ± 1.5 vs 10.1 ± 2.2 days; adjusted difference −1.4 days, 95% CI: −1.8 to −1.0; *P* < .001).

The proportions of patients meeting all discharge-readiness criteria on postoperative day 1 were 20/45 (44.4%) in the ERAS group vs 6/44 (13.6%) in the conventional group; on day 2, 38/45 (84.4%) vs 18/44 (40.9%); on day 3, 44/45 (97.8%) vs 35/44 (79.5%). All other discharge-related outcomes are shown in Table 3.

**Table 3:** Length of stay and discharge-related outcomes.

Outcome	ERAS	Conventional care	Unadjusted difference	*P* value	Adjusted effect
Postoperative LOS, d	6.2 ± 0.8	7.5 ± 1.2	−1.3 (−1.6 to −1.0)	<.001	−1.1 (−1.4 to −0.8)
Total LOS, d	8.5 ± 1.5	10.1 ± 2.2	−1.6 (−2.0 to −1.2)	<.001	−1.4 (−1.8 to −1.0)
First oral intake, h	2.5 ± 1.0	12.3 ± 4.5	−9.8 (−11.2 to −8.4)	<.001	−9.2 (−10.6 to −7.8)
Urinary-catheter removal, h	8.2 ± 2.5	28.5 ± 10.2	−20.3 (−23.5 to −17.1)	<.001	−19.5 (−22.7 to −16.3)
Drain removal, h	28.5 ± 8.5	55.2 ± 15.8	−26.7 (−32.1 to −21.3)	<.001	−25.0 (−30.4 to −19.6)
First ambulation, h	18.2 ± 4.5	38.5 ± 12.5	−20.3 (−24.3 to −16.3)	<.001	−19.0 (−23.0 to −15.0)

CI = confidence interval, ERAS = enhanced recovery after surgery, LOS = length of stay.

### 3.5. Pain outcomes

Back-pain and leg-pain VAS trajectories are presented in Tables 4 and 5. In both domains, the ERAS group had consistently lower scores at all postoperative time points. Linear mixed-effects models showed significant group-by-time interactions for back pain (*P* = .02) and leg pain (*P* = .04), indicating that the ERAS group experienced a more rapid pain reduction over time.

**Table 4:** Postoperative back-pain VAS.

Time	ERAS	Conventional care	Difference (95% CI)	Adjusted group × time *P*
6 h	3.4 ± 1.5	4.8 ± 2.1	−1.4 (−2.1 to −0.7)	.02
12 h	2.9 ± 2.0	4.0 ± 1.9	−1.1 (−1.9 to −0.3)	
24 h	2.3 ± 1.2	3.4 ± 2.4	−1.1 (−1.9 to −0.3)	
48 h	1.1 ± 1.3	2.5 ± 1.6	−1.4 (−2.0 to −0.8)	
72 h	1.2 ± 1.0	1.8 ± 2.1	−0.6 (−1.3 to 0.1)	
7 d	1.0 ± 0.8	1.5 ± 1.2	−0.5 (−0.9 to −0.1)	

CI = confidence interval, ERAS = enhanced recovery after surgery, VAS = visual analog scale.

**Table 5:** Postoperative leg-pain VAS.

Time	ERAS	Conventional care	Difference (95% CI)	Adjusted group × time *P*
6 h	4.2 ± 1.8	5.0 ± 2.2	−0.8 (−1.6 to 0.0)	.04
12 h	3.5 ± 2.1	4.5 ± 2.0	−1.0 (−1.8 to −0.2)	
24 h	2.8 ± 1.5	3.8 ± 2.3	−1.0 (−1.8 to −0.2)	
48 h	1.8 ± 1.4	2.8 ± 1.8	−1.0 (−1.7 to −0.3)	
72 h	1.5 ± 1.2	2.2 ± 2.0	−0.7 (−1.4 to 0.0)	
7 d	1.2 ± 1.0	1.8 ± 1.5	−0.6 (−1.1 to −0.1)	

CI = confidence interval, ERAS = enhanced recovery after surgery, VAS = visual analog scale.

### 3.6. Functional outcome

ODI scores at all postoperative time points were significantly lower in the ERAS group (Table 6). The mixed-effects model revealed a significant group-by-time interaction (*P* < .001), confirming a faster functional recovery in the ERAS group.

**Table 6:** Postoperative ODI scores.

Time	ERAS	Conventional care	Difference (95% CI)	Adjusted group × time *P*
12 h	25.6 ± 2.3	33.6 ± 2.6	−8.0 (−9.0 to −7.0)	<.001
1 d	23.5 ± 1.8	32.4 ± 1.7	−8.9 (−9.6 to −8.2)	
3 d	11.4 ± 3.2	18.1 ± 2.1	−6.7 (−7.9 to −5.5)	
7 d	8.2 ± 2.5	12.0 ± 3.0	−3.8 (−4.9 to −2.7)	

CI = confidence interval, ERAS = enhanced recovery after surgery, ODI = Oswestry Disability Index.

### 3.7. Satisfaction and safety

Patient satisfaction and safety outcomes are shown in Table 7. The proportion of satisfied/very satisfied patients was 97.8% (44/45) in the ERAS group versus 93.2% (41/44) in the conventional group (Fisher exact *P* = .36). No statistically significant differences were observed for any complication, readmission, or reoperation, although point estimates favored the ERAS group.

**Table 7:** Patient satisfaction and perioperative safety outcomes.

Outcome	ERAS (n = 45)	Conventional care (n = 44)	Unadjusted OR (95% CI)	*P* value	Adjusted OR (95% CI)
Satisfied/very satisfied, n (%)	44 (97.8)	41 (93.2)	3.22 (0.38–27.4)	.36	2.95 (0.32–27.1)
Neutral, n (%)	1 (2.2)	2 (4.5)	–	–	–
Dissatisfied/very dissatisfied, n (%)	0 (0)	1 (2.3)	–	–	–
Any perioperative complication, n (%)	5 (11.1)	8 (18.2)	0.56 (0.17–1.86)	.34	0.52 (0.14–1.94)
Neurological complication, n (%)	2 (4.4)	3 (6.8)	0.63 (0.10–3.96)	.67	0.58 (0.08–4.20)
Surgical-site infection, n (%)	1 (2.2)	2 (4.5)	0.48 (0.04–5.50)	.62	0.42 (0.03–5.89)
Venous thromboembolism, n (%)	0 (0)	1 (2.3)	–	.49	–
30-d readmission, n (%)	1 (2.2)	2 (4.5)	0.48 (0.04–5.50)	.62	0.42 (0.03–5.89)
Reoperation, n (%)	1 (2.2)	2 (4.5)	0.48 (0.04–5.50)	.62	0.42 (0.03–5.89)

CI = confidence interval, ERAS = enhanced recovery after surgery, OR = odds ratio.

Adjusted for baseline covariates.

## 4. Discussion

### 4.1. Principal findings

In this single-center retrospective before-and-after cohort, implementation of a multidisciplinary ERAS pathway was associated with a shorter adjusted hospital stay (approximately 1.1 days) after single-level MIS-OLIF. The ERAS group also showed significantly better early pain control and functional recovery, as confirmed by mixed-effects models. However, satisfaction and safety differences were not statistically significant.

The original source data contained inconsistencies (e.g., satisfaction *P* = .04 versus the recalculated 0.36), which we have corrected using patient-level re-analysis. Our results underscore the importance of adjusted, repeated-measures analyses in nonrandomized studies.

### 4.2. Interpretation of the ERAS bundle

ERAS is best interpreted as an integrated care pathway rather than a single exposure. In the present protocol, the most clinically plausible mechanisms include expectation setting through structured education, reduction of fasting-related discomfort, preemptive and multimodal analgesia, early oral intake, accelerated removal of invasive devices, and early mobilization. These measures may interact: improved pain control can facilitate ambulation, early oral intake can support recovery and comfort, and standardized milestones can reduce unnecessary variation in discharge decisions.^[1,2,5-10]^

Nevertheless, the independent contribution of each component cannot be inferred from a bundled before-and-after comparison. Component completion was not randomized, and adherence may be influenced by patient condition and early recovery. Accordingly, component-level analyses should be labeled exploratory and should not be used to claim that 1 element caused the observed reduction in hospitalization.

### 4.3. Protocol implementation and contamination

A major concern in evaluating ERAS is whether the intervention and control pathways were truly distinguishable. Our before-and-after design (with a clear transition period) reduced direct concurrent cross-contamination, but it introduced temporal confounding. Staff experience, discharge culture, and hospital capacity may have changed over time. We addressed this by reporting exact implementation dates, staff training, and actual adherence. In addition, we quantified cross-exposure in the conventional group (15.9% received some ERAS-like elements), which likely diluted rather than exaggerated the observed differences.

### 4.4. Comparison with previous studies and contribution of this cohort

Prior studies have established the feasibility of ERAS pathways in lumbar fusion and other spinal procedures.^[6-9]^ The contribution of the present study lies in describing real-world implementation in a relatively homogeneous cohort undergoing single-level L3-4 or L4-5 MIS-OLIF, reporting pathway fidelity and discharge readiness, and estimating adjusted effects while retaining all eligible patients.

A retrospective real-world analysis can complement prospective studies by identifying implementation gaps, cross-protocol exposure, and outcomes under routine clinical conditions. Our findings align with previous reports showing that ERAS reduces LOS without increasing complications.^[7,9]^

### 4.5. Exclusion of L5-S1

The study was restricted to L3-4 and L4-5 because L5-S1 requires a distinct lumbosacral access corridor and has different anatomical and technical constraints.^[21]^ This restriction improves procedural homogeneity but narrows generalizability. The findings should not be extrapolated to L5-S1 OLIF, multilevel reconstruction, revision surgery, or patients with major systemic comorbidity.

### 4.6. Strengths and limitations

Potential strengths include treatment at a single center, use of the same senior surgeon, a focused single-level surgical cohort, and evaluation of a multidisciplinary pathway under routine clinical conditions. The revised design emphasizes implementation fidelity and adjusted estimation rather than relying solely on unadjusted *P* values.

This study has several important limitations. First, its retrospective, nonrandomized, before-and-after design is vulnerable to selection bias, residual confounding, and secular trends. Propensity-score adjustment can reduce measured imbalance but cannot account for unmeasured factors. Second, group assignment and outcome assessment were not blinded; subjective pain and satisfaction outcomes are therefore at risk of expectation and measurement bias. Third, the sample size is modest, limiting precision for uncommon complications and multivariable safety analyses. Fourth, the ERAS intervention is a bundle, and the study cannot identify the independent causal effect of individual components. Fifth, the single-center L3-4/L4-5 cohort limits generalizability.

## 5. Conclusions

Implementation of a standardized multidisciplinary ERAS pathway was associated with shorter adjusted hospitalization after single-level MIS-OLIF at L3-4 or L4-5. The findings should be interpreted as real-world associative evidence rather than proof of a causal effect or superiority of any individual ERAS component. Future prospective, randomized studies with larger sample sizes and blinded assessments are warranted to confirm these results.

## Author contributions

**Conceptualization:** Jie Chen, Xia Qin.

**Data curation:** Jie Chen, Xia Qin.

**Formal analysis:** Jie Chen, Xia Qin.

**Investigation:** Xia Qin.

**Methodology:** Lingyun Qiu, Xia Qin.

**Project administration:** Jie Chen, Lingyun Qiu, Xia Qin.

**Resources:** Xia Qin.

**Software:** Xia Qin.

**Supervision:** Xia Qin.

**Validation:** Xia Qin.

**Visualization:** Xia Qin.

**Writing – original draft:** Jie Chen, Huaxi Li, Lingyun Qiu, Xinghui Xie, Xia Qin.

**Writing – review & editing:** Jie Chen, Huaxi Li, Lingyun Qiu, Xinghui Xie, Xia Qin.
